# Eighteenth Annual Session of the Mississippi Dental Association

**Published:** 1892-07

**Authors:** 


### THE DENTAL REGISTER.

Vol. XLVI.] JULY, 1892. [No. 7.

### Proceedings.

### Eighteenth Annual Session of the Mississippi State Dental Association, Columbus, May 3, 4, and 5, 1892.

The Mississippi State Dental Association convened its Eighteenth Annual Session, in Concordia Hall, Columbus, Tuesday, May 3, 1892.

The Association was called to order by Dr. W. W. Westmoreland, Chairman of the Executive Committee, and opened with prayer by the Rev. J. L. Johnson, Pastor of the Baptist Church.

Officers present:

Dr. D. B. McHenry, Grenada, President.

Dr. A. A. Dillehay, Meridian, First Vice President.

Dr. N. N. Wofford, Columbus, Third Vice President.

Dr. W. E. Walker, Bay St. Louis, Recording Secretary.

Dr. P. H. Wright, Senatobia, Corresponding Secretary.

Drs. W. W. Westmoreland and N. N. Wofford, Columbus, and Dr. P. H. Wright, Senatobia, Executive Committee.

Dr. Westmoreland introduced Col. S. M. Meek, of Columbus, who delivered an eloquent address of welcome. Rarely has such a noble tribute to the rapid advancement, and the scientific standing of the dental profession been paid. After alluding briefly to his surprise that he, a lawyer, should have been selected to welcome the Dental Association, he said as he ever felt a lively interest in whatever adds to the culture, advancement and elevation of any one of the learned professions, it was therefore with sincere pleasure and unalloyed gratification that he undertook the task of extending to one and all, individually and collectively, a warm and hearty welcome to the hearts, homes and firesides of the residents of Columbus.

After a glowing and well-merited tribute to the claims of Columbus to her honored name, a city which, in the words of a noted scholar, "looks like a little city that had gone on a pic-nic, and camped for a time, amid forest trees and flowers;" with her broad and well-shaded streets, handsome residences and beautiful flower-gardens, houses of worship of all denominations, noble educational institutions, and great manufacturing establishments; her active and energetic population, the men cultured and refined, her women gentle, fascinating and beautiful, he spoke of the great benefits of associated effort in any occupation, calling or profession, as being the great hour and hand guide for the accomplishment of good.

The dental profession, based upon the loftiest principles of art and science forming no exception, but forcing its way to solid strength and permanent prosperity, through the combined power of associated effort.

Reviewing the past history of dentistry, Col. Meek said that he had been astonished in the reading and investigation he had given the matter, in attempting to prepare himself for this occasion, to see the lofty pinnacle to which it has risen; until to-day, its members are almost countless, its magazines, periodicals, journals, and other publications being weekly, if not daily, issued by the press; dental schools and colleges are springing up in nearly all the States of the Union; laws have been found for the direction, regulation and control of those desiring to enter its ranks, and the prospects before it are gratifying and encouraging. Thus, he said, safeguards are abundantly thrown around the profession, and the public cannot be imposed upon by incompetent men if the Board of Examiners perform their duty faithfully and well. Blacksmiths, silversmiths and jewelers must stick to their callings, and not tamper and tinker with that of which they know nothing. Quacks, pretenders and charlatans must and will stand aside if this Board, appointed by the Governor—men eminent for their learning and ability—meet the demands of a confiding public.

To this eloquent address of welcome, Dr. R. K. Luckie, of Holly Springs, the retiring President of the Association responded, in language equally fluent and eloquent. He spoke of the rejoicings which filled the hearts of all when Columbus was selected as the next place of meeting. He said that all Mississippians were naturally proud of Mississippi, with her vast, undeveloped domain, inviting the agriculturist with promise unequaled, a climate almost matchless, a soil as fertile as the valley of the Nile, with millions of acres of virgin forests—with schools, colleges, churches and missions, all in a prosperous condition. He paid a well-merited tribute to Columbus—as a city of fragant flowers, of taste and refinement, of beautiful homes, of great men and noble christian women—a city of historic associations and traditions, worthy to stand in perpetual memory of the most adventurous navigator that ever braved the ocean's waves, a heroic man, a man of scientific, philosophic, religious faith, of indomitable perseverance under intimidating difficulties; a man who dared to stand before kings and advocate a project decided as visionary. He lamented the too-well-known lack of enterprise in the South, which for so many years has left the manufacture of the great staple—cotton—in the hands of the Old and New England, whereby millions have been lost annually; and expressed his gratification that Columbus had taken the initiative in starting this great industry. The cotton mills of Columbus having proved a great financial success, having won an enviable reputation for high-grade products. He spoke in eloquent terms of the "Industrial Institute and College" of Columbus, a State institution for girls; he said that, though Mississippi had no gold, no silver, no copper, no rubies, sapphires nor diamonds, yet no country in the world had jewels of more priceless value than the pure and lovely girls of that institution; jewels being polished by the State, and fitted to beautify christian homes and brighten human hopes. He then spoke of the Mississippi State Dental Association, and portrayed its growth and progress from the date of its birth, April 21, 1875, through eighteen years of varying fortunes, standing well the test of time. Through its influence, wholesome dental laws have been enacted, dental education has been encouraged. Through associated effort, its members have been stimulated to the highest efforts in scientific investigations and in the introduction of new methods of work in all departments—the progressive dentist of to-day being prouder of his calling than he has ever been. Advances are being made all along the line; scientific investigations are more thorough; new discoveries are being made; new instruments, materials and appliances are being introduced; the standard of dental education has been elevated, and there is constant desire for higher, nobler and better things. Good things have come; better things are yet to come; better, not only for the profession, but for the public at large. We can truthfully say no one can foretell the future of our loved and honored profession.

Dr. Westmoreland next introduced Dr. D. B. McHenry, of Grenada, the President of the Association, who proceeded to deliver the

ANNUAL ADDRESS.

This, though brief, dealt with many points of practical importance to the association as a body, and to the individual members. He urged the adoption of some plan whereby members might come prepared to intelligently discuss the papers presented; the more active work of the members appointed in each judicial district; to see to the enforcement of the dental law of the State; the discussion of, and the means of securing changes in the dental law somewhat surreptitiously passed by the last legislature, and which, as it now stands, antagonizes both the Association and the Board of Examiners, and the appointment of a committee to look after the interests of the dental profession of the State of Mississippi, at the Columbian Dental Congress and in co-operation with the local members of the Special Committees.

After a short recess for roll call and payment of dues, on motion of Dr. Westmoreland, the Lowndes County Medical Association, in session in Columbus, were invited to seats in the Dental Association with the privilege of discussion.

During the sessions, Drs. Brownrigg, Vaughn and James availed themselves of the invitation, adding greatly to the interest of the discussions.

Prof. Francis Peabody, of the Dental Department of the University of Kentucky, Louisville, honorary member of the

Association, was introduced, and made some remarks in explanation of an instrument or appliance to be used in his clinics, in the treatment of root canals; the invention of Prof. John C. Blair, demonstrator of operative dentistry in the Dental Department of the University of Kentucky. The instrument consists of a small cylinder with a rubber bulb as in a syringe; the cylinder being filled with crystals of iodoform and heated over a lamp, the iodoform is sublimed, the fumes, by pressure on the bulb, being forced through the nozzle into the roots of teeth filled with putrescent pulp, destroying all forms of germ life, overcoming one of the difficult features in the treatment of root canals. The instrument is new, and its present form crude, and the odor of the fumes of iodoform forms an objection to its use in the office, but it offers great possibilities in experimenting with other germ-destroying agents, perhaps menthol. In the Infirmary of the Louisville College of Dental Surgery, it has been used in three or four hundred cases which would otherwise have been condemned to the forceps; only one case, treated by a student, proving unsatisfactory, and in that, it was found that the root canal had not been opened into. Teeth which were extracted, after being treated in this manner, on being ground down on the lathe were found to be perfectly permeated with iodoform, even through the tubuli to the peridental membrane. The iodoform vapor thus introduced is deposited in crystals to the very apex of the root canal, forming a desirable permanent filling material at that point, as it is soluble only in ether or chloroform. It has been found, in Dr. Blair's experience of two years with it, very valuable in the treatment of blind abscess, of loose teeth, and in teeth that are too tender and sore to be treated by the old methods. In one case after ten days usual treatment, of a very loose, inferior bicuspid, with no diminution of soreness or swelling; one application of this new treatment, by Dr. Blair, cured all soreness and swelling, and at the end of six days, the tooth was as sound as any other tooth in the jaw. Another case given was that of a superior lateral incisor, which was filled with the debris of a decomposed pulp, and had given trouble for four years; a single application subdued all irritation, and the tooth was filled immediately after the deposition of iodoform crystals from the vapor. In all cases Dr. Blair removes all debris, applies the vapor and fills immediately. He has had but one failure in two years. In conclusion, Dr. Peabody stated that his own personal experience with the new method had been limited; this "was not his baby, he was only its nurse." He did not want to overlook its imperfections, but thought that in a future modified form it offered great possibilities.

Owing to some misunderstanding in the notification of Chairmen of Sections, but one section was prepared with papers—that of Operative Dentistry, which was called at the night session of the first day. The Chairman, Dr. Geo. B. Clement, (Macon, Miss.,) responded with an oral discourse, explanatory of a series of diagrams which he displayed, illustrating various types of incisor tooth forms; also similar teeth mounted in plaster, in support of a theory as to "Primary Causes of Decay," found in defects in enamel structure. He said that it was now universally admitted that it was of prime importance that the mouth, being the entrance of the alimentary canal, should be pure, clean and healthy. It seemed paradoxical that the teeth, though the hardest tissue of the organism should be the first to decay, while the brain, the softest tissue of all, is the last to decay. It is rare to find a human being with absolutely perfect teeth. As dentists we are called upon to repair defects and restore them to normal utility. It should be our work to prevent these defects, but patients usually come to us when it is too late for preventive measures.

Dr. Clement's first chart represented the lingual surface of incisors in which were seen certain fissures—defects in enamel structure, affording points where decay first starts. In a chart of the labial surfaces, similar defects were seen at the junction of the enamel and the cementum where a crevice would frequently be found. The natural defects are the initial or primary causes of decay, and all such fissures and crevices, it is claimed by Dr. Clement, should be cut out and filled in the best manner with gold, thereby preventing decay, which will otherwise be inevitable through the lodgement of particles of food, the formation of acids, and the generation of germ life. We do not want to wait until the patient is suffering from extensive ravages of decay. The approximal surfaces of teeth decay at the points of contact through pressure and the friction of natural lateral movements. Three bicuspids, imbedded in plaster were passed around in support of this theory; one had a slight fissure, another a small cavity, the third a large carious cavity. Dr. Clement claimed that if the second and the third tooth had been filled when in the condition of the first, the caries of their present condition would have been prevented. When there is no fissure, but only an abrasion caused by friction and pressure, the teeth may be separated, and the surface only polished. He said that cutting out and filling a fissure is an aseptic operation; if delayed, the use of antiseptics becomes necessary. The diagrams represented three types of incisor teeth—one where they touch only at the incisor edge, with a narrow base at the gum margin; in this type we will find a small point of decay at the point of contact. Another type has a broad base touching only at the cervical margins where also we will find decay, due to pressure, though food will lodge higher up. A third type consisted of square, straight sided teeth; these touch the whole length of the sides, and if pressed apart with rubber, initial points of decay will be found all along the sides. Whether we adopt the germ theory or the acid theory, makes no difference in the facts; wherever continuity of tooth surface is interrupted decay is invited, even the slightest defect, which the eye, or even the instrument may not be able to detect. In perfect tooth form the external shape indicates the shape of the pulp cavity. In the narrow base tooth we are very liable, in excavating, to strike the pulp. In the bicuspids we are liable to touch the horns of the pulp. All defects or fissures should be excavated at once and filled with gold; all abrasions should be separated and polished.

In the discussion of the subject, Prof. Crenshaw, of the Dental Department of the Southern Medical College, Atlanta, Ga., Honorary Member of the Association, said that the square teeth represented in the diagram, and the narrow based incisors touching only at the cervical margins, instead of being typical tooth forms, were very unusual except as the result of slashing and filing.

Dr. Clement admitted that they were somewhat overdrawn, to illustrate his theory.

Dr. Crenshaw said that the speaker was correct as to the correlation between the external shape of the teeth and the shape of the pulp cavity.

By the study of the typal forms of teeth, we are enabled to make more satisfactory fillings, and especially to avoid the horns of the nerves.

Dr. Westmoreland, (Columbus,) thought we should give more study to the prevention of decay; that children should be safeguarded in embryo, so that they would come to us with good teeth. He considered eruptive diseases in childhood a great source of defective tooth structure and consequent decay. The defects in the labial aspect of the incisors, pointed out by Dr. Clement, were in nine out of ten cases caused by eruptive diseases occuring while the teeth were forming.

Prof. R. R. Freeman, (Nashville, Tenn.,) of the Dental Department of the Vanderbilt University, and Honorary Member of the Association, wished to endorse the words uttered as to the early care of the teeth, this being the greatest importance as to refinement, purity and healthfulness. From the time there is but one tooth, watchful care must be taken to secure cleanliness. If food was kept thoroughly cleaned out, there would be no chemical abrasion—acids are carried by capillary attraction to the point of contact. He is so thoroughly convinced of this necessity of thorough cleanliness that he puts across the corner of his bill-heads the motto, neither original or new, but always true, "Clean teeth don't decay." It will not do to begin at three years of age, nor at six months; the work must begin, or should have been begun three generations before the child was born. Little children forget to brush their teeth, that is true, but not more than to wash their faces or brush their hair; parents should look after the former as they do after the latter. The secretions in the mouth of a child are acid, as evidenced in the green stain, but this must be removed, and its renewal prevented by proper care. It is not the persons of most robust development that live the longest, but those who take the greatest care as to how they live. It is so with the teeth. It is not those that have the best structural development that will last the longest, but those of which care is taken. Establish correct habits in the earliest years of a child's life, and the work is done.

Dr. A. A. Dillehay asked as to the causes of the decay when alkaline fluids predominated.

Dr. Clement replied that normal mouth fluids are always alkaline, but that local acids cause the trouble.

Dr. W. E. Walker, Bay St. Louis, read a paper on "Practical Anæsthetics." A synopsis follows:

He spoke of the dental profession as being eminently humanitarian, that while called upon to relieve pain, or to give prophylactic treatment, yet, in the very nature of the case, in many of the operations to be performed, pain is an inevitable factor, except by the employment of the preventive means found in the judicious use of anæsthetics. After briefly reviewing the evidences of the use of anæsthetics or hypnotic agents by the ancients; and the discovery of modern anæsthetics, that proud boast of the dental profession, Dr. Walker spoke first of hypnotism as an anæsthetic agent. He said, if the half that is told be true, we would seem to have in hypnotism, a valuable adjunct to dental practice. If without any interference with pulse or respiration, such complete insensibility to pain can be induced by the mere will of the operator, that the severest and most painful tests can be applied to the unconscious patient, why not extract teeth, or operate upon the dental pulp? The Dental Review, in July, 1890, republished from the medical news, the report of an interesting trial of hypnotism as an anæsthetic agent, employed by Milne Bramwell, in the presence of upwards of sixty medical practitioners and dentists. Among other painless operations, three teeth were extracted for one woman and sixteen stumps removed for another, the latter not even experiencing any subsequent soreness of the gums or mouth. At a meeting of the Chicago Anæsthetic Club in October, 1890, Prof. Anderson, of Denmark, lectured on this subject, and gave a number of severe tests.

At a subsequent meeting of the same club, Prof. Norman J. Roberts gave a clinic with hypnotic demonstrations before an audience of physicians and dentists among whom were mentioned, Drs. L. P. Haskell, Jno. S. Marshall, J. J. R. Patrick, and others well-known to the dentists of Mississippi. Many severe tests were applied, respiration and pulse remaining normal, as tested by a physician present. Prof. Roberts predicts that hypnotism will, within a few years, take the place of ether and chloroform, specialists being employed to hypnotize patients for all operations when anæsthesia is desired.

Dr. Walker said that he would confine his paper to the consideration especially of Local Anæsthetics; first—because the operations the dentist is most frequently called upon to perform are not such as would either *require* or *justify* the use of general anæsthetics, not only because of the risk and inconvenience attending their exhibition, but also because of the frequent impracticability of securing proper antecedent conditions of the patient, and secondly, because the operation for which an anæsthetic is most frequently demanded—that of extraction of teeth—is so often for transient patients with whose constitutional idiosyncracies and organal pecularities we are not familiar.

In the treatment of *sensitive dentine*, Dr. Walker relies upon warm air gently applied, with encouraging sympathetic words and with gentle firmness. Herbst's obtundent—a saturated solution of cocaine hydrochlorate in chemically pure sulphuric acid, to which solution sulphuric ether is added to the point of saturation, often gives very satisfactory results. The application of ether spray causes pain quite as severe as the excavation of the cavity, excellent results are claimed for the combination, 10 parts chloroform, 15 parts ether, 1 part menthol, used in the hand atomizer. He also quoted Dr. Geo. Earnes instructions: "put on the dam, secure perfect dryness by using warm air, and apply cocaine dissolved in chloroform in a 10 per cent. solution;" also Dr. Littig's formula: "a saturated solution of cocaine in glycerine," as being worthy of trial.

For the *removal of the pulp* with little or no pain, Dr. Walker has used successfully Dr. A. W. Harlan's preparation, a solution of 10 grs. hydrochlorate of cocaine in 90 minims of sulphuric ether, left in contact with the pulp for five minutes.

For the *extraction of teeth*, Dr. Walker early adopted the use of cocaine. To secure a reliable aqueous solution, he adopted the plan of having a number of small vials, each containing such a definite quantity, by weight, of hydrochlorate of cocaine crystals that the addition of distilled water, up to a file mark on the vial at the time of using, gave ten drops of a fresh 4 per cent. solution without any delay or uncertainty. This, however, he said, was obviated by the introduction of Parke, Davis & Co.'s hypodermic tablets, one of which dissolved in ten drops of water, gives as much of the 4 per cent. solution as it is advisable to use "above the shoulders."

After citing a number of authorities on the use and risks of cocaine and antidotes, and relating some incidents in practice, Dr. Walker spoke of his experience with the following formula, given to the dental profession, about a year ago, by L. C. Wasson, of Topeka, Kansas, through the Kansas State Dental Association. Dr. Wasson had received it from Dr. Guibor, a specialist in the treatment of diseases of the nose and throat:

| | |
|---|---|
| Cocaine hydrochlorate | gr. 20 |
| Sulphate of Atropia | gr. 1-10 |
| Carbolic acid crystals | gr. 10 |
| Chloral hydrate | gr. 5 |
| Aqua pura add | one oz. |

Although the maximum hypodermic dose of this preparation, two syringes full or 60 minims, for extensive operations, contains 2 3-10 gr. cocaine, the hypodermic dose being ¼ to 1 gr. the whole making a 6 1-5 per cent. solution of cocaine, yet it is so combined with antidotal and localizing agents that its use is much less objectionable than cocaine alone, in a 2 per cent. or a 4 per cent. solution in much smaller quantity.

Of the other elements, Dr. Wasson says, "(1) atropia in small doses, as given in hypodermic injections from this preparation is a cardiac, respiratory and spinal stimulant, which tends to contract the toxic effects of the cocaine; (2) carbolic acid aids the chloral in localizing the anæsthesia, and both tend to increase the anæsthetic properties of the cocaine, and localize its effects, while (3) both aid in the preservation of the solution, which is, in itself, quite desirable, as the ordinary cocaine mixture is almost worthless at the end of a week, while this preparation is good for months." Dr. Walker said that he had used this preparation very extensively for hypodermic injections in the gum tissue, for the past eight months, and had found it more satisfactory than anything he had used, especially for tooth extraction. Though he had been very cautious in his previous experience with cocaine, never to inject more than ¼ gr., yet he frequently found it necessary to use the proper antidotes to combat toxic symptoms; yet with this preparation, though he frequently injected 30 minims, containing from 1 to 1 1-6 grs. cocaine, he had not had one case requiring any antidote, and only three cases of very slight systemic effects, scarcely worthy of the name. These three cases he described in detail. He cautioned, however, as to one unpleasant feature sometimes attending the injection; where the quantity injected is very large, and the gum tissue very dense and unyielding slough may follow, confined, however, strictly to the area subjected to pressure. He had had three cases of this nature, only one of which threatened to become serious. The superior central incisors being very badly decayed, it was necessary, in using a Perry Separator, to press severely and high up on the gum; this caused so much pain that the injection was resorted to with very satisfactory results at the time. The operation was rendered painless; but a slough supervened which being posterior to the incisors on the median line threatened possible rupture of the anterior palatine vessels with probably severe hemorrhages. Fortunately it did not extend so deep, and healed without any serious results.

In conclusion, Dr. Walker regretted that he had not been able to carry out his intention of experimenting with cocaine phenate as a local anæsthetic. According to Merck's Bulletin "its practical insolubility in water is a physical property most favorable to its therapeutic action, as it cannot be readily washed away from its site of application by the lymph currents. Its local action is therefore more intense and prolonged. In consequence of its insolubility very small doses have effect, while, on the other hand, excessively large doses (15½ gr.) are not prone to produce toxic symptoms; this makes it of value in topical applications—as by the brush—where the dosage cannot be regulated with exactness."

In a private letter received from Merck's Laboratory since reading the above paper: Dr. Walker is further informed that the percentage of cocaine alkaloid in cocaine phenate is about 75 per cent.. That it is soluble in alcohol of from 30 to 50 per cent., and that as an anæsthetic in dental operations it produces complete topical anæsthesia without subsequent derangement of the general system. An alcoholic solution containing 1 part of the drug in 1250 of alcohol was the form employed.

Dr. Brownrigg, practicing physician of Columbus, being requested to open the discussion, said that he felt diffident in speaking on the specialty of dentistry, a profession whose labors add to the longevity of race; but that in the able and interesting paper to which he had listened with pleasure, he noticed that ether had been omited, and he wished to enter a plea in its behalf. He hoped they would investigate its merits as an anæsthetic and try it. That chloroform was very objectionable, many of the great hospitals having repudiated its use. But he considered sulphuric ether entirely harmless; it could be used with perfect safety. He had employed it largely for twenty-seven years, and almost exclusively for nineteen years. Full anæsthesia is not necessary in minor operations, analgesia is all that is necessary. Operations in dentistry are of such an awakening nature that the patient awakens readily, and there is no subsequent trouble from blood in the mouth. The very short time required to produce analgesia with ether should make it popular with dentists when time is very valuable. Cocaine is undoubtedly valuable, but it has its risks and dangers, while ether is absolutely harmless.

Dr. Walker stated that he had not included ether nor any other general anæsthetic in his paper which he had purposely limited to local anæsthetics as being practicable and all-sufficient for the average minor operations of the dental surgeon.

Mr. Foster, in charge of the Dental Exhibit from the S. S. White Atlanta Depot, asked the privilege of the floor that he might speak of the merits of chloride of ethyl as a local anæsthetic.

Dr. Westmoreland stated that Dr. James, a local physician, was an expert in the art of hypnotism, and would give a clinic the following day.

Dr. W. H. Marshall, (Oxford,) had used chloride of ethyl to a limited extent, but found that it would not keep in a temperature above 60°. Had been able to excavate the most sensitive dentine painlessly, also found ½ tube sufficient for painless extraction.

Dr. R. R. Freeman wished to take exception to the idea expressed in the paper that dentists were not as fully acquainted with the constitutional idiosyncracies of their patients as the physician. It should always be remembered that dentists introduced anæsthesia, and we should uphold the honor of our profession. He was glad that ether had been spoken of; that he both used and liked it, but did not class it as absolutely harmless. Nature gives pain as a protection, and he wants the extraction of teeth to be painful as a safe-guard against its ruthless practice. Though he does not employ the subtle force of hypnotism yet he believes the dentist may show such force of character and power of command, that the patient will yield to the inevitable; some might perhaps call it hypnotism. Any person can anæsthetize himself by rapid breathing or rather blowing; when a patient will do this for a few minutes, the smallest amount of ether in a very few inhalations will suffice for the extraction of even four or five teeth. But in its use we should impress upon our patients that we are taking into our hands an agent that requires extreme care. "Beware how you handle it." He said that he would withstand the temptation of all the gold of Ophir rather than permit the use of chloroform in his office. He regarded the use of nitrous oxide gas as similar in effect to plunging a persons' head in a bucket till on the verge of drowning, and then extracting his teeth—only there was no water to pump out! But the appearance of the person in either case would be equally repulsive. His experience with cocaine had been such that he did not want any more of it; it excites hysteria and other evil influences. Pure water, or even "make believe," is enough if you have control over the mind of the patient.

Dr. Walker said that he hoped he was not generally so misunderstood as that he would belittle his profession. He had the highest possible opinion of the attainments of dentists, and was far from denying them the privilege of administering general anæsthetics, but there were too many preliminary requirements; we would often have to send them home for looser garments; we must have a third party present, and this was not always convenient. As to not understanding the constitution, etc., of our patients, the patients whom we do understand, and who are under our care, are not as a rule those for whom we extract teeth; we generally save their teeth. But those for whom we do the most extracting are "transients" whom we have perhaps never seen before, and may never see again.

Prof. Peabody wished to go on record as saying that no systemic anæsthetic known to-day is absolutely free from danger; whether it be ether, chloroform, nitrous oxide gas or cocaine. If Dr. Freeman was capable of producing anæsthesia with rapid breathing and sulphuric ether in one, two, or three minutes, he must have sufficiently strong powers of hypnotism to make them believe he was operating painlessly. He had used sulphuric ether for the extraction of teeth, but he had known cases where he had used half a pint of ether, and taken thirty minutes to do it, without producing sufficient anæsthetic effect to operate painlessly. With the various temperaments and idiosyncracies of individuals different effects are produced upon different people, and on a stranger no one can say what the effect will be. The most skilled physicians cannot. With the known average of one death in 1,000 from chloroform, one in 10,000 from ether, and one in 100,000 from nitrous oxide, we are not justified in resorting to them for minor operations. There is a well-known case where a lady had been under her physician's care for fifteen years; he had safely administered chloroform to her sixteen times, the seventeenth time she died from it. It is true that in the sixteen times when it had been administered safely, she was prone upon a table, while the seventeenth time she was reclining in a chair; but that is where our patients usually are. "No sum of money would induce me to allow it used in my office, though I would use it for the extraction of teeth at the patient's residence with the physician present." As to rapid breathing, he considered it one of the most trying experiences he had ever undergone, and he doubted if any person could breathe one hundred times in a minute. Nitrous oxide gas acts by asphyxiation, it cuts off the nerves of sensation. If a person will draw in a long breath four or five times, and then fill the lungs to their fullest capacity and hold their breath, the most sensitive cavities can be excavated. For the extraction of teeth, the patient may hold the breath till the critical moment, but then the inevitable *cough* will expel the breath, and the effect is lost. So-called "Christian Science" is nothing but fanaticism; it is not christian, it is not scientific; it is the old story of the influence of mind over matter. The subject of anæsthesia is one of the greatest interest. It should be generally known that, notwithstanding the report of the Commission of Hyderabad, India, that we are liable with chloroform to have paraylsis of the cardiac muscles, that sulphuric ether affects both the heart and respiration; that nitrous oxide gas is an asphyxiating agent. If cocaine is used, have ammonia and nitrate of amyl at hand. In Europe the use of these things is denied to the dental surgeon, they are not considered capable of understanding these things, even though endowed with the same amount of brains as the general surgeon; perhaps it is thought that there is something in the study of dentistry that renders the brain torpid! But, whether physician or dentist, the use of these agents is never unattended with danger, and therefore each one should thoroughly familiarize himself with the action of each.

Dr. Walker said that he was much gratified that his paper had called out these criticisms and this discussion, as it has been said that "it takes a good paper to call out a good discussion."

But we must bear in mind that accidents, sometimes fatal, occur even at the hands of physicians who have known their patients all their lives. Our own patients whom we know as well, want their teeth saved. For "transients" there is less risk in a local anæsthetic, which with less time, less trouble and less inconvenience, fills all the needs of the occasion.

On motion of Dr. R. K. Luckie, (Holly Springs,) the discussion was closed and the subject passed.

The second day was devoted to clinics.

Previous to the night session of the second day, Dr. E. P. James, of Columbus, gave an interesting clinic in "hypnotism," though no person was found for a dental operation under this influence, a pin was passed through the ear of the subject, and other remarkable tests given.

At the night session, second day, a letter was read from Dr. Gordon White, President of the Southern Dental Association, giving a cordial invitation to all members of the Mississippi State Dental Association, to attend the meeting of the Southern Dental Association at Lookout Mountain, July 26, 1892.

On motion of Dr. Westmoreland, Dr. R. R. Freeman was elected an Honorary Member of the Association.

On motion of Dr. A. H. Hilzim, of Jackson, members were put in nomination, and the five following, having received the highest number of votes cast, were declared the choice of the Association for recommendation to the Governor of the State, for appointment on the new Board of Dental Examiners, according to the amended Dental Law passed by the last Legislature, viz:

Dr. W. E. Walker, Bay St. Louis; Dr. B. Clements, Macon; Dr. Geo. Rembert, Natchez; Dr. P. H. Wright, Senatobia; Dr. J. Warriner, Corinth.

Dr. B. A. Vaughn, a practicing physician of Columbus was introduced to the Association, and addressed them in the interests of two bills now pending before U. S. Congress.

At the close of his address the following resolutions were introduced and carried, and ordered sent to the State Senators and Representatives in Congress:

*Resolved*, That we approve of and urge the passage of the bill creating a Department of Public Health;

*Resolved*, That we approve of the bill "Pure food," and urge its passage.

There being no paper from the section of "Prosthetic Dentistry," the subject in general was discussed at some length.

Dr. D. B. McHenry, Grenada, President of the Association, presented a new device for partial plates, which he terms the "Skeleton Lock Plate." This is a narrow plate, strengthened by an imbedded gold band, and retained firmly in place by the insertion in a posterior tooth, on either side, of a pin with rounded projecting head, under which the plate springs. It cannot tip or fall out, though it is easily removed by the patient. It forms an inexpensive substitute for bridgework, and does away with the objectionable grinding down and dressing of the natural teeth necessary for the caps and crowns of bridgework.

Prof. R. R. Freeman, Professor of "Mechanical and Corrective Dentistry" in the Dental Department of Vanderbilt University, expressed himself as fully repaid for the trip in seeing this device alone, which he considered well worthy the attention of every dentist. He said that he was slow to pick up new devices, but he should certainly adopt this one.

Of the "Anchor Plate" presented to this Association last year by Dr. W. H. Marshall, of Oxford, Dr. Freeman said that he had found it one of the needful things. It had bridged over great difficulties in the adaptation of partial plates. Though very simple in construction, it accomplishes all that is claimed for it by the inventor. In some cases one, and in other cases, the other of these two new methods will be found the one thing needed—*par excellence.*

Drs. Nesbit, Hilzim and Spinks, who have been inventing "Anchor Plates" during the past year, spoke highly of the great satisfaction the method has given, especially where suction plates have been found objectionable. Dr. Spinks had found it of the greatest service, when a suction plate could not be worn because of paralysis. Also in another case where a patient had been debarred from his greatest pleasure, flute-playing, for nineteen years, while wearing a suction plate; but who, with the "Anchor plate," found himself able to play the flute with the same ease, as when he had his natural teeth.

At the morning session of the third day, some amusing incidents of office practice were related by Drs. Westmoreland, Walker, and others.

Drs. Spinks and Dillehay gave the details of a serious case of Necrosis following the extraction of a tooth, which led to a very full discussion of the treatment of Necrosis.

Prof. Peabody said that it was difficult to state the cause of Necrosis unless the patient knew of some direct injury. In case of depraved blood, impoverished pabulum, a very slight injury might be followed by most serious results. He was very pronounced in favoring a surgical operation, cutting well beyond the diseased parts, for the thorough removal of every portion of infected tissues. When vitalized tissue is reached, the mouths of the vessels are open and pabulum will flow in. He would always leave a ring of gum tissue around the necks of loose teeth, leaving them in place if possible even when removing the septi and alveolar process, the periosteum of live bone should always be preserved; sub-periosteal bone—the sub-osteoblasts—being the great source of osteo genesis. Bibulous paper folded many times and packed in the wound will prevent infiltration of the buccal fluids and prevent septic irritation. A cure is the result of thorough manipulation, and the maintenance of aseptic conditions. He said that in the case of pyorrhea alveolis, the margins of the sockets were always necrosed and should be removed to hasten absorption; otherwise we would have to wait for the solution of the dead portions. Absorption would be much slower, though it would eventually be greater.

Dr. Clement wonders that we do not have more cases of Necrosis, for every time we extract a tooth we open into cancellous structure, and there are from nineteen to twenty-two varieties of micro-organisms ready to infiltrate into the cavity. He would resort to surgical operation if the disease had invaded the cancellous portions between the inner and the external plates of the maxillary; but would rely on medication with aromatic sulphuric acid, if only the margins of the alveolus were affected.

A thorough operation, with dissection of all the soft tissues demands a thorough knowledge of the anatomy of the parts, but if we know where the nerves and blood vessels are located, we can avoid them and operate safely.

Dr. Freeman, on the other hand, thought the brave, wise and judicious man was he who knew how to keep his hands off, and let nature do her work properly, watching the tendency of the pathology of the condition, whether towards degeneracy or regeneration. If the latter wait and only assist nature, she will rally to the performance of her functions. There is always a question in heroic surgery if we have gone far enough. If we had waited we would not have added fuel to the flames. We open up a new field for the infiltration of bacteria, and enlarge the scope of their devastations. If we have patience to wait, in a large number of cases nature's benign influence will work a cure, and we will not be forced into these bloody operations of surgery. Use the proper precautions to "fight the bugs" and nature will work the cure.

The several advantages of dilute pure sulphuric acid, and aromatic sulphuric acid were discussed at length. Dr. Freeman advocating dilute sulphuric acid as taught by Dr. Wm. H. Atkinson, spices having no agency in reproducing bone tissues, or in getting rid of dead bone.

Dr. Peabody urged the tonic effects of the spices added in the aromatic preparation.

Dr. Clement also favoring the latter for its stimulating action.

Dr. Vaughn being appealed to, gave it as his opinion that the spices were added simply to obtund the pain caused by the pure acid, and to make it more acceptable.

The subject being passed, Dr. R. K. Luckie, member of the State Committee, addressed the Association on the subject of the Dental Congress of the World's Columbian Exposition, reading the suggestions as to lines of work offered by Dr. Taft, Chairman of the General Committee of Conference. He set forth very clearly the objects and aims of the Dental Congress, and urged upon each and all the duty of contributing to the funds of information desired in compiling a history of dentistry throughout the world.

Dr. Peabody, of Kentucky State Committee, and Dr. Freeman, of the Tennessee State Committee, seconded the address of Dr. Luckie.

There being no more papers, and no other business, the election of officers was held, with the following result:

Dr. A. A. Dillehay, Meridian, President.

Dr. A. A. Wofford, Columbus, First Vice President.

Dr. L. G. Nisbit, Aberdeen, Second Vice President.

Dr. W. T. Allen, Amory, Third Vice President.

Dr. W. E. Walker, Bay St. Louis, Recording Secretary.

Dr. Frank H. Smith, Grenada, Corresponding Secretary.

Dr. C. C. Crowder, Kosciusko, Treasurer.

Jackson was selected as the next place of meeting, carrying it by one vote over Pass Christian on the gulf coast.

The Dental Law as recently amended, provides for the meeting of the State Board of Dental Examiners at Jackson, the first week in April. That date was accordingly fixed for the meeting of the Association.

The usual Resolutions of thanks were passed, bills paid, the officers elect installed in office, and on motion the Association adjourned to meet in Jackson, Wednesday after the first Tuesday in April, 1893.

